# Does CO_2_ emissions–economic growth relationship reveal EKC in developing countries? Evidence from Kazakhstan

**DOI:** 10.1007/s11356-019-06166-y

**Published:** 2019-08-17

**Authors:** Fakhri J. Hasanov, Jeyhun I. Mikayilov, Shahriyar Mukhtarov, Elchin Suleymanov

**Affiliations:** 1 King Abdullah Petroleum Studies and Research Center, P.O. Box 88550, Riyadh, 11672 Saudi Arabia; 2 Research Program on Forecasting, Economics Department, The George Washington University, 2115 G Street, NW, Washington, DC 20052 USA; 3 Institute of Control Systems, B. Vahabzade Street 9, AZ1141 Baku, Azerbaijan; 4 Department of Statistics and Econometrics, Azerbaijan State University of Economics, Istiqlaliyyat Str., 6, AZ1001 Baku, Azerbaijan; 5 Institute for Scientific Research on Economic Reforms, 88a, Hasan Bey Zardabi Avenue, AZ1011 Baku, Azerbaijan; 6 Department of World Economy, Baku Engineering University, Hasan Aliyev 120, AZ0101 Khirdalan, Azerbaijan; 7 UNEC Empirical Research Center, Azerbaijan State University of Economics (UNEC), Istiqlaliyyat Str. 6, AZ1141 Baku, Azerbaijan; 8 Department of Finance, Baku Engineering University, Hasan Aliyev 120, AZ0101 Khirdalan, Azerbaijan; 9 The Institute of Economics, Azerbaijan National Academy of Sciences, H. Javid pr., 115, AZ1001 Baku, Azerbaijan

**Keywords:** CO_2_ emissions, Economic growth, EKC hypothesis, Cointegration, Kazakhstan, C32, Q01, Q43, Q52, Q53, Q56

## Abstract

This paper investigates the CO_2_ emissions–economic growth relationship in Kazakhstan for the period 1992–2013. Johansen, ARDLBT, DOLS, FMOLS, and CCR cointegration methods are used for robustness purpose. We start with the cubic functional form to rule out any misleading results that can be caused by misspecification. Although the estimation results suggest “U”-shaped relationship, the turning point of income is out of the period. It means that the impact of economic growth on CO_2_ is monotonically increasing in the long run indicating the Environmental Kuznets Curve (EKC) hypothesis does not hold for Kazakhstan. Moreover, we calculate that the income elasticity of CO_2_ is about unity. The paper concludes that the Kazakhstani policymakers should focus on less energy-intensive sectors as well as using more renewable energy in order to avoid higher pollution effects of economic growth. They may also set new policy regulations for CO_2_ reduction.

## Introduction

Environmental degradation-related studies have been gaining increasing importance and popularity since the pioneering papers by Grossman and Krueger (1991), Shafik and Bandyopadhyay (1992, SB hereafter), and Panayotou (1993). The importance of these studies gains special strength considering the fact that the 2 °C increase of the global temperature can cause inevitable and unsolvable problems for the residents of our common globe (Nordhaus 1975). As Nordhaus (1975) mentioned “… carbon dioxide will probably be the first emission to affect climate on a global scale, with a significant temperature increase by the end of the century.” In this regard, many developed as well as developing countries signed the Kyoto protocol in 1997, to protect the nature and avoid the fatal consequences of uncontrolled energy use and economic development. At the beginning, the protocol put emission reduction requirements for developed countries. However, later on, it turned out that the developing countries have an increasing share in global emissions (Winkler et al. 2002). The level of CO_2_ emissions (CO_2_ hereafter) from developing countries has been rapidly exceeding that of developed countries, which was almost 50% of the world’s CO_2_ in 2003 (Martínez-Zarzoso and Maruotti 2011). The significant share among the developing countries, in terms of environmental degradation, belongs to resource-rich, oil-exporting countries. Since these countries have rich natural resources (such as oil, gas, and coal) and cheap/subsidized prices for them, the focus on the economic development might cause unnecessary and uncontrolled use of endowment and as a consequence can end up with the significant climatic deteriorations. In this regard, the investigation of CO_2_–economic growth relationship in case of the abovementioned countries gains special significance. Due to the facts listed previously, the current study analyzes the emission–income relationship for the 11th country in the world, in terms of proven oil reserves, the second largest oil producer among the former Soviet countries in 2014 (KCCG 2016), and the 9th largest country in terms of land area—Kazakhstan. Kazakhstan’s economy benefits from its natural resources (particularly oil, gas, coal, and uranium), heavy industry, and agricultural sectors. The petroleum and mining industries accounted for 33% of GDP in 2010 and 82% of exports (NRGI 2014). Kazakhstan’s GDP increased 13.3 times from 16.9 billion USD in 1999 to 224.4 billion USD in 2013 (ASRK 2013). Approximately 87% of Kazakhstan’s power is generated from thermal-powered plants (75% coal-fired stations and 12% gas-fired plants) (Kadrzhanova 2013), which are considered as main contributors of CO_2_. According to the World Bank (2007), CO_2_ stemming from the burning of fossil fuels and the manufacture of cement is responsible for almost 60% of greenhouse gas (GHG). The energy sector in Kazakhstan is responsible for carbon dioxide emissions of 275 Mt CO_2_ in 2011 with 80% derived from the energy sector from heat and power generation (UNFCCC 2013). In addition, as other industrialized post-Soviet countries during almost the soviet century, Kazakhstan has not considered either in Baikonur polygon or in other industry sectors environmental and/or ecological problems as a main concern in the development path because targeting industrialization and communism in the USSR is the focus than the other main problems. In this regard, the findings of the current research are important not only for Kazakhstan but also for the other victims of the same ideological system.

In order to protect the environmental quality and avoid the consequences of uncontrolled economic development in terms of negative impact on the climate, the Kyoto Protocol (KP) was signed by the Kazakh government in 1999 and ratified in 2009 (Reuters 2009). The ratification of the KP was followed by the law of “On Amendments to Certain Legislative Acts of the Republic of Kazakhstan Relating to Environmental Issues” in 2010. Introducing this law intensified the country’s capability to take part in carbon markets. Kazakhstan started domestic emission trading system (ETS) in 2012 to achieve the target of reducing greenhouse gas (GHG) emissions at 7% below the 1990 levels by 2020 (PETER 2014). The Kazakh ETS’s plan is to operate similar to the European Union’s ETS. The Kazakh’s ETS targeted to contribute to the country’s beforehand identified emission reduction commitments (IETA 2014). Ever since, Kazakhstan has been implementing the number of aforementioned mitigation measures, and the results of the realized policies need to be accessed using the proper measurement techniques.

Considering all the above-mentioned facts, the investigation of the CO_2_–income relationship in the case of Kazakhstan gains special importance. To the best of our knowledge, Akbota and Baek (2018) is the only time series study focusing solely on Kazakhstan rather than group of countries. However, it has a number of weaknesses. It appears that there is no time series study for Kazakhstan that employs appropriate functional forms and different cointegration methods. Hence, the objective of the current study is to model the CO_2_–income relationship in Kazakhstan, analyze the features of this relationship, and provide appropriate policy insights.

The study uses time series data ranging from 1992 to 2013 and employs different cointegration methods for robustness purpose. It found that there is a long-run relationship between CO_2_ and income. We conclude with the U-shaped form with the turning point being outside the sample meaning that the income has a positive impact on CO_2_. This shows that the Environmental Kuznets Curve (EKC) does not hold for Kazakhstan. Moreover, we assess that the income elasticity of CO_2_ is about unity.

The contributions of the study are that, to the best of our knowledge, it is a first time series study devoted to CO_2_–income modeling in Kazakhstan with the following features: (a) It employs the functional form suggested by the seminal studies rather than restricting itself with quadratic or linear functional form. Few earlier panel studies tested the EKC hypothesis in Kazakhstan using linear and quadratic functional forms and ended up with the results, which are not consistent with the conventional common sense of EKC for the developing economies. To avoid such potential misleading consequences, we started with the cubic functional form as suggested by SB among others. (b) We are not aware of any time series studies, except one, that focus purely on Kazakhstan, not group of countries, employ appropriate functional forms, perform robustness check using different methods, and address small sample bias correction. Only Akbota and Baek (2018) purely focuses on Kazakhstan. However, we have some concerns about these studies. We do not discuss the concerns here as the next section reports them. The third contribution of the study is to revisit the interpretation of the coefficients of the polynomial functional form, which appeared in the current literature in an improper way—interpreting the coefficients of the powers of the same variable separately.

The novelty of the current study is that it investigates the CO_2_ emissions–economic development relationship in the case of oil-exporting developing country case to see and discuss the contradicting findings for such countries in the so-called EKC literature. As expected, the study concludes invalidity of EKC for the Kazakhstan case, as a developing country. The finding of EKC phenomenon for the developing country cases might be due to the following reasons: (a) The use of improper functional specification. (b) Misinterpretation of the results. That is, interpretation of the results without referring to the potential cases discussed in seminal papers. An example to this case might be the interpretation of the “U-shaped” relationship, which should be interpreted as an “N-shaped” one, since for the impact of economic development on CO_2_ to be negative first it is expected to be negative up to some threshold level of income. Another example can be the interpretation of the empirically found “inverted U-shaped” curve without investigating the situation of the turning point. As known if the turning point is out of the used span, bigger than the maximum point, this finding should be interpreted as monotonically increasing relationship. (c) The use of unsettled data span. In other words, as it is known in econometrics for the data as well as relationships among the variables, as if a car started to move from the inertia situation, it takes some time to be settled and get the long-run path. If one uses the data for which the relationship is not settled down s/he most likely will end up with misleading results. (d) The use of improper proxies for the used indicators. As an example, CO_2_ emissions can be proxied by consumption- and production-based ones, and these measures can work differently depending on the country case. (e) Not taking into account the country-specific features, such as the unreliability of the reported data, or not having the clear picture of socio-economic development path of the investigated country. This list can be expanded with some other points as well in future research. The point we wanted to emphasize here is that the finding of the EKC in developing country case requires caution and should be investigated further.

The main policy recommendation of the study is that Kazakhstani policymakers should consider that future economic growth will result in more CO_2_. Therefore, three sets of measures seem to be important—focusing more on the less energy-intensive sectors in the economic development, increasing the share of renewables in energy generation, and setting new regulations to reduce CO_2_.

## Literature review

The main focus of this section is to review the studies devoted to the emissions–income relationship in Kazakhstan. However, such studies are very limited, and, hence, we additionally review the studies for the country group, in which Kazakhstan is included. Table 1 summarizes the studies.

**Table 1:** Review of CO_2_ studies for Kazakhstan

Study	Sample	Country or region	Functional form	Econometric methodology	Data	Income elasticity	Shape of EIR
Tamazian and Rao (2010)	1993–2004	24 transition economies including Kazakhstan	QLF	GMM	PD	0.04–1.22 ln GDP^a^	IUS
Apergis and Payne (2010)	1992–2004	11 CIS countries including Kazakhstan	QLF	FMOLS	PD	For panel with Russia: 1.55–2.96 ln GDP; For panel without Russia: 1.37–2.54 ln GDP^b^	IUS
Stolyarova (2013)	1960–2008	93 countries including Kazakhstan	LLF	GMM	PD	Short-run elasticity: 0.3–0.79^c^	Not reported
Brizga et al. (2013)	1990–2010	15 former Soviet countries Kazakhstan	LLF	Index decomposition analysis and OLS	TD	0.86	Kazakhstan IUS
Perez-Suarez and Lopez-Menendez (2015)	1860–2012	175 countries including Kazakhstan	CLF	NLS	TD	Not reported	No specific pattern
Narayan et al. (2016)	1960–2008	181 countries including Kazakhstan	Correlation coefficients are used	Cross-correlation estimate	TD	Not reported	Kazakhstan US
Al-Mulali et al. (2016)	1980(1990)-2010	107 countries including Kazakhstan	QLF	DOLS	PD	4.75–0.18 ln GDP for the group with Kazakhstan^d,e,f^	IUS for the group with Kazakhstan
Erdoğan and Ganiyev (2016)	1992–2013	Central Asia including Kazakhstan	QFF	Fixed effect and random effect	PD	0.13	IUS
Ito (2017)	2002–2011	42 developing countries including Kazakhstan	LLF	GMM and PMG	PD	GMM: 0.13 PMG: 0.34	MI
Mitic et al. (2017)	1997–2014	17 transitional economies including Kazakhstan	LLF	DOLS and FMOLS	PD	0.35	MI
Shuai et al. (2017)	1960–2011	164 countries including Kazakhstan	QFF	OLS	TD, PD	0.81–0.08 ln GDP	IUS
Akbota and Baek (2018)	1991–2014	Kazakhstan	QFF	ARDL	TD		IUS

^a^For different specifications, the coefficients are slightly different; hence, we took the average of obtained coefficients (results from Table 4 were used) and calculated the elasticity.

^b^The mean of ln GDP is not provided; hence, only elasticity formula is calculated

^c^QLF is used, but, then, the squared term is excluded due to multicollinearity

^d^Only the model with growth rates is used

^e^Authors’ calculation is based on the results of that study

^f^Started with CLF and concluded with LLF

EIR, emission–income relationship; ARDLBT, autoregressive distributed lag bound testing; FMOLS, fully modified ordinary least squares; DOLS, dynamic ordinary least squares; GMM, generalized method of moments; OLS, ordinary least squares; PMG, pooled mean group estimator; NLS, non-linear least square method; PD and TD, panel data and time series data; CIS, commonwealth of independent states; MI, monotonically increasing; US, U-shaped; IUS, inverted U-shaped; LLF, log-linear function; QLF, quadratic functional form in logarithm; CLF, cubic functional form in logarithm

Modified from Mikayilov et al. (2018)

Many of the studies in the table are the panel studies. It is hard to draw a proper picture of CO_2_–income relationship for Kazakhstan based on the panel studies because of the well-known weaknesses of the panel analysis (Hsiao 2003; Kasprzyk et al. 1989).^1^ There are only four time series studies but only Akbota and Baek (2018) focused solely on Kazakhstan.^2^ We appreciate greatly these studies as they are only the ones conducting time series analysis for Kazakhstan. At the same time, and for the sake of preventing readers from any misperception of CO_2_–income relationship for Kazakhstan, we would like to point out our concerns on these studies. Shuai et al. (2017) conducted panel and time series analyses for 164 countries, including Kazakhstan. Hence, not enough attention was paid to the country-specific issues. For example, the study reports that it uses data from 1960 to 2011 for the countries including Kazakhstan. However, Kazakhstan got its independence in 1991 and prior to that it was under the Soviet Union. It means that the authors put together two different systems’ data for dependent and explanatory variables. This may lead to a serious issue called measurement error, which makes regression coefficient and standard hypothesis testing invalid if it is observed in the explanatory variable of a regression (see Hayashi 2000; Gujarati and Porter 2009 inter alia). Besides, the authors restrict themselves by using the quadratic functional form rather than starting with more unrestricted, i.e., cubic form, and thus might be subject to functional misspecification problem. Moreover, the study uses OLS although it finds cointegration for Kazakhstan. The issue is that standard inferential statistics are invalid for the OLS-based results even though the variables are cointegrated (Park and Phillips 1988). Furthermore, the study concludes that the EKC hypothesis holds for Kazakhstan, which is hard to believe as the hypothesis usually holds true for developed/advanced economies. In fact, the authors find that the per capita income level for the turning point of the relationship is far away from the sample values, meaning that monotonically increasing relationship between emissions and income level prevails although the quadratic functional form is specified (see Cole et al. 1997; Stern and Common 2001 for discussion).

**Fig. 1:**
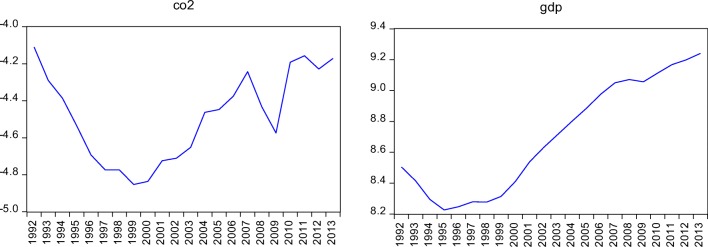
Time profile of the variables (in logarithmic form)

The following are our concerns for Akbota and Baek (2018), in particular, their finding of IUS for Kazakhstan, which would not seem realistic as it is usually the case for advanced economies. First, they restrict themselves by using the quadratic functional form. Meaning that if the CO_2_–income relationship in Kazakhstan follows higher polynomial than quadratic then the study suffers from the functional misspecification problem. Second, the study’s main finding is that CO_2_ and income have a positive relationship until 2001 and the growing income is associated with the decreasing CO_2_ since then. This finding contradicts with Fig. 1 and endnote 4 in the paper. Third, a number of seminal scholars in the EKC field recently argue that it is not correct to include energy consumption variable in CO_2_–income relationship and calculate turning point (e.g., see Itkonen 2012; Liddle 2015; Jaforullah and King 2017). It is good that the authors acknowledge this issue. However, they do not address the issue in complete coverage.^3^ Fourth, on the one hand, the study found that income and its quadratic term are trend-stationary variables; on the other hand, it discussed that ARDLBT can be applied when regressors are I(1) or a mix of I(1) and I(0). Enders (2015) among others explains that it is not appropriate to take a difference of the trend-stationary variables in modeling and forecasting. Fifth, it would be suggestive to perform robustness check using more than one cointegration methods and report the results as the study finds something, which would not seem reasonable for Kazakhstan.^4^

Table 1 shows that only Perez-Suarez and Lopez-Menendez (2015) examined emissions–income relationship starting with the cubic functional form for Kazakhstan.

One can conclude from the literature review here that there is no time series study devoted specifically to Kazakhstan, which starts with the cubic functional form to rule out misspecification and uses different cointegration methods and small sample bias correction to robust obtained results. We address all the mentioned issues in this research.

## Theoretical framework and data

### Employed functional form

In estimations, we start with the cubic functional form to rule out potential misspecification issue (SB; Grossman and Krueger 1995; Lieb 2003; Dinda 2004, inter alia):

1 $$ {co}_{2t}={b}_0+{b}_1{y}_t+{b}_2{y}_t^2+{b}_3{y}_t^3+{b}_4{x}_t+{u}_t $$

where *co*_*2*_ is *CO*_*2*_ per capita, *y* is *GDP* per capita, *x* is a vector of additional explanatory variables, and *u* is the error term. Often Eq. (1) is estimated with a time trend in order to capture the effects of technological progress or enhance environmental awareness on CO_2_ (SB; Lieb 2003).

Due to space limitation, we do not discuss the details and different hypothesis testing using Eq. (1) here. They are discussed in Mikayilov et al. (2018).

### Data

We used an annual time series data on CO_2_ measured in kilotons (kt) of carbon dioxide and gross domestic product per capita measured in US dollars at 2010 prices over the period 1992–2013 for Kazakhstan taken from the World Bank Development Indicators Database 2016 release (WB 2016). Note that selection of the period is based on data availability: GDP per capita is available until 2015 whereas CO_2_ is available only up to 2013. CO_2_ per capita is calculated using population data from WB (2016).

Figure 1 illustrates the time profiles of the natural logarithm levels of CO_2_ per capita and GDP per capita, denoted by CO_2_ and gdp, respectively, over the period 1992–2013.

The CO_2_ per capita decreased more than 2 times in Kazakhstan for the period 1992–1999. This decrease can be explained by different factors, such as the shutdown or weakening of the industrial sector after the collapse of the Soviet Union. For 2000–2013, the relative increase with some volatility and drops in 2008 and 2009 can be observed in the time profile of the variable. This increase (and decrease in some cases) can be explained by the implemented energy policies over the period.

As a general tendency for GDP per capita during the chosen period, it has increased persistently since 1999. The variable was decreasing in each year of 1992–1995 due to the collapse of the Soviet Union and thus the centrally planned economic system and other related issues. The growth rates of the variable turned to positive in 1996 and 1997 but were negative in 1998 mainly caused by the Russian crisis. Due to higher oil prices during the 2000s coupled with a dominant share of oil and gas sectors in the Kazakhstani economy, the GDP per capita increased persistently although it was negatively impacted by the global financial crisis in 2008 and 2009. The economy and thus GDP per capita recovered again as oil price and global oil demand recovered and raised significantly.

Table 2 presents descriptive statistics of the *co2* and *gdp* for 1992–2013.

**Table 2:** Descriptive statistics of the variables

Variable	Minimum	Mean	Maximum	Standard deviation	Coefficient of variation (%)
*co2*	− 4.85	− 4.48	− 4.11	0.24	5.36
*Gdp*	8.23	8.70	9.24	0.36	4.14

## Econometric methods

Note that we employed the natural logarithm expressions of CO_2_ per capita (*co2*) and GDP per capita (*gdp*). Our empirical analysis will cover the following stages. First, we will check non-stationary characteristics of the variables.^5^ We will use the augmented Dickey–Fuller unit root test (Dickey and Fuller 1981, ADF hereafter) for this examination. We will also employ the Kwiatkowski–Phillips–Schmidt–Shin (Kwiatkowski et al. 1992, KPSS hereafter) test to increase the robustness of our inference. Note that KPSS takes the null hypothesis of stationarity (or trend stationarity) while all other conventional univariate unit root tests, including the ADF, take the null hypothesis of the unit root. We do not discuss the tests here due to the space limitation, and interested readers can refer to Dickey and Fuller (1981), Kwiatkowski et al. (1992), and Enders (2015).

Second, if the integration orders of the variables are the same, then we will apply cointegration test(s) to see whether they are cointegrated. We will use the Johansen test (Johansen 1995) as it is the only test that can produce proper results in the case, where more than two variables are tested for cointegration.

Third, if we find only one cointegrated relationship among the variables, then alongside the Johansen method, we will also use other alternative cointegration and long-run estimation methods to increase the robustness of our inferences on the long-run relationship. For this purpose, we will use the single equation-based cointegration method, which is autoregressive distributed lag bound testing (ARDLBT hereafter) developed by Pesaran and Shin (1999) and Pesaran et al. (2001) as it outperforms all the alternative cointegration methods in small samples. As further robustness, we will use Narayan (2005) critical values in the ARDLBT alongside Pesaran et al. (2001) critical values for the purpose of the small sample bias correction. We will also employ dynamic ordinary least squares (DOLS), fully modified ordinary least squares (FMOLS), and canonical cointegrating regression (CCR), which are based on the residual-based cointegration method developed by Engle and Granger (1987).

We do not describe the above-mentioned methods here in order to save space and to not bother readers with econometric discussion.

## Empirical results

This section documents the results of the empirical analysis.

### Unit root test

Table 3 reports the ADF and KPSS unit root test results.

**Table 3:** The UR test results

Variable	The ADF test					The KPSS test				
	Test value	*C*	*t*	*None*	*k*	Test value	*C*	*t*	*None*	
*co2*	− 2.71	x	x		0	0.18**	x	x		
*Gdp*	− 2.53	x	x		1	0.13*	x	x		
*gdp*^*2*^	− 2.52	x	x		1	0.14*	x	x		
*gdp*^*3*^	− 2.50	x	x		1	0.14*	x	x		
Δ*co2*	− 1.97**			x	2	0.44*	x			
Δ*gdp*	− 2.67*	x			2	0.37*	x			
Δ*gdp*^*2*^	− 2.65*	x			2	0.39*	x			
Δ*gdp*^*3*^	− 2.63*	x			2	0.40*	x			

Maximum lag order is set to two and optimal lag order (*k*) is selected based on the Schwarz criterion in the tests. The critical values for the ADF and KPSS tests are taken from MacKinnon (1996) and Kwiatkowski et al. (1992), respectively. Estimation period 1992–2013. None means that neither intercept nor trend is included in the test equation. Note that unit root test equation can include one of the following: intercept (*C*), intercept and trend (*t*), and none of them (*None*). x indicates that the corresponding option is selected in the equation

**Indicates rejection of the null hypotheses of unit root in the ADF and stationarity or trend stationarity in the KPSS at 5% significance level

*Indicates rejection of the null hypotheses of unit root in the ADF and stationarity or trend stationarity in the KPSS at 10% significance level

The ADF sample values, reported in Table 3, suggest that all the four variables are I(1). In other words, the levels of the variables contain unit root and thereby are non-stationary while the first differences of them are stationary. The sample statistics of the KPSS also support the conclusion that the variables are non-stationary at the level and stationary at the first difference. For example, at the level test, KPSS rejects the null hypothesis of trend stationarity at the 10% significance level for the per capita income and its quadratic and cubic terms. Meaning that if we go for the 10% significance level, considering that we have the small number of observations, the income variables are the unit root processes.^6^ Our finding that the variables are I(1) is expected as usually economic and energy/environmental indicators are non-stationary at their levels and stationary at their first differences. Moreover, our findings are in line with the EKC literature.

### Estimation results from the Johansen approach

As discussed in the “Theoretical framework and data” section, we will start with the cubic functional form in order to avoid any misleading which can potentially be caused by using the restricted functional form.

Following the estimation procedure of the Johansen method, a VAR with endogenous variables of *co2*, *gdp*, *gdp*^*2*^, and *gdp*^*3*^ and exogenous variables of intercept, trend, and one pulse dummy are specified.^7^ We estimate the VAR with two lags, as we did in the unit root test exercise since it can provide uncorrelated residuals, a key issue in VAR estimations (see Johansen 1990, 1995 among others). Panels A through C in Table 4 report that the VAR is stable and its residuals have no issues with serial correlation and non-normal distribution.

**Table 4:** The VAR residual diagnostics and cointegration test results

Panel A: serial correlation LM test^a^					
Lags	LM statistic	*P* value			
1	17.63	0.35			
2	11.22	0.80			
3	23.37	0.10			
Panel B: normality test^b^					
Statistic	χ^2^	d.f.	*P* value		
Skewness	5.48	4	0.24		
Kurtosis	6.07	4	0.19		
Jarque–Bera	11.55	8	0.17		
Panel C: stability test^c^					
Modulus	Root				
0.96	0.91 − 0.29i				
0.96	0.91 + 0.29i				
0.67	0.18 − 0.64i				
0.67	0.18 + 0.64i				
Panel D: Johansen cointegration test summary					
Data trend	None	None	Linear	Linear	Quadratic
Test type	(a) No *C* and *t*	(b) Only *C*	(c) Only *C*	(d) *C* and *t*	(e) *C* and *t*
Trace	4	3	1	2	1
Max-Eig	2	2	1	1	1
Panel E: Johansen cointegration test results for type (*c*)					
Null hypothesis	*r* = 0	*r* ≤ 1			
*λ*_trace_	59.85***	29.26*			
*λ*_max_	30.59**	16.91			

^a^The null hypothesis in the serial correlation LM test is that there is no serial correlation at lag order *h* of the residuals

^b^System normality test with the null hypothesis of the residuals is multivariate normal

^c^VAR stability test results show that no roots of characteristic polynomial are outside the unit circle

^*^Rejection of null hypothesis at 10% significance level

^**^Rejection of null hypothesis at 5% significance level

^***^Rejection of null hypothesis at 1% significance level

χ^2^, Chi-squared; d.f., degree of freedom; *C* and *t,* intercept and trend; *r*, rank of Π matrix, i.e., number of cointegrated equations; *λ*_trace_ and *λ*_max_, trace and max-eigenvalue statistics

Critical values for the cointegration test are taken from MacKinnon et al. (1999); estimation period 1994–2013

As next step of the Johansen method, we perform the cointegration test on the transformed version of the VAR, which is VECM with one lag order. Evidently from panel D, both the trace and max-eigenvalue statistics indicate the same number of cointegrated relationship, which is one, in only the test types of (c) and (e).^8^ Therefore, we do not discuss the results from other test types. Moreover, we think that test type (e), where a quadratic trend is included in cointegration equation of *co2*, would not be reasonable as *co2* did not demonstrate any quadratic trend pattern either graphically or in the unit root test. Thus, we focus on test type of (c) and numerical values of the statistics for this type are presented in panel E. Note that such focus is also consistent with the conventional preference in the EKC literature. Panel A of Table 5 shows the long-run equation for *co2* in type (c).

**Table 5:** The long-run equations

Panel A: Cubic functional form: *co*2 = *α*_0_ + *α*_1_*gdp* + *α*_2_*gdp*^2^ + *α*_3_*gdp*^3^ + *e*				
	*co2* = 2124.13 −	704.57*gdp* +	*77.49gdp*^2^ −	2.83*gdp*^3^ + *e*
		(453.63)	(52.30)	(2.01)
Panel B: Quadratic functional form, when *α*_3_ = 0				
χ^2^ (1) = 1.71 [0.19]	*co2* = 151.20 −	36.13*gdp* +	2.09*gdp*^*2*^ *+ e*	
		(7.96)	(0.46)	

Values in bracket and parentheses are probability and standard errors, respectively. Estimation period 1994–2013

Evidently, from panel A, *gdp*^3^ is highly insignificant. We believe that this insignificancy makes the coefficients of *gdp* and *gdp*^2^ statistically insignificant and very large. In fact, we restrict the coefficient on *gdp*^3^ to zero and the *Chi-squared* test statistic in panel B shows that the restriction cannot be rejected. Meaning that the restriction is statistically significant and valid. Panel B also reports results from the restricted long-run equation, which is the quadratic functional form now. Now, the magnitude of coefficients on *gdp* and *gdp*^2^ is reasonable and highly significant. The only issue with the quadratic functional form is that SoA is positive and statistically insignificant. We think that this is because of the following two facts: (a) we have only 20 observations against four endogenous variables with two lags for each in the estimation and (b) although we restricted *gdp*^3^ to zero, it is still in the cointegration space with zero coefficient. Therefore, as a next step, we exclude *gdp*^3^ from the VAR/VEC analysis and replicate all the steps that we did in the case of the cubic functional form. The lag order and exogenous variables in the VAR of the quadratic functional form appear as the same as it was in cubic functional form. The VAR also successfully passes all the stability and residual diagnostic tests. Test type of (c) produces more reasonable cointegration results when we conduct the Johansen cointegration test. All the mentioned estimation and test reports can be obtained from the authors upon request due to the space limitation. Another merit of the VAR is that now SoA coefficient is − 0.03 although it is still not significant at the conventional level, which is caused by the small sample. Estimated long-run coefficients of the VEC transformation of the VAR are reported in Table 7 alongside the coefficients from other alternative methods for comparison purpose.

### Robustness check for the long-run relationship

This sub-section presents the robustness check for the long-run relationship obtained from the Johansen approach. We conclude that the Johansen cointegration test suggests one long-run relationship among the variables. This allows us to employ ARDLBT as well as DOLS, FMOLS, and CCR for further analyzing the long-run relationship of *co2* as a robustness check.

We first estimated cubic functional form using the four long-run methods discussed previously. The results are the same as what we got from the Johansen method. Precisely speaking, the cubic term is statically insignificant from all the four methods.^9^ We exclude *gdp*^*3*^ and estimate quadratic functional form using the methods. We give priority to the ARDLBT and discuss it a little bit more as it outperforms all its counterparts when the sample size is small, which is the case for our research here. We set the maximum lag order of two in the ARDL estimation, being consistent with what we did in the VAR/VEC estimation.^10^ Then, we consider the Schwarz information criterion to find optimal lag orders for each variable. The only *ARDL(1,1,1)* specification succeeds as it does not have any problem with the serial correlation, non-normality, heteroscedasticity, ARCH effect, and misspecification. Table 6 summarizes the ARDLBT estimation and test results.

**Table 6:** The ARDLBT estimation and test results

Panel A: Selected ARDL specification					
$$ co{2}_t={\theta}^{\prime }+\sum \limits_{i=1}^1{\alpha}_i^{\prime } co{2}_{t-i}+\sum \limits_{i=0}^1{\beta}_i^{\prime }{gdp}_{t-i}+\sum \limits_{i=0}^1{\gamma}_i^{\prime } gd{p^2}_{t-i}+{\delta}^{\prime }\ trend+{e}_t $$					
Panel B: Residual diagnostics and misspecification test results for *ARDL*(1,1,1)					
${\chi}_{SC}^2(2)$=2.35 [0.31]	${\chi}_{ARCH}^2(2)$*=*2.51 [0.29]	${\chi}_{HETR}^2(6)$*=*4.16 [0.65]	*JB*_*N*_*=*0.74 [0.69]		*F*_*FF*_*=*0.71 [0.42]
Panel C: The cointegration test results for *ARDL*(1,1,1)					
The sample *F*-statistic	Significance level (%)	Pesaran et al. (2001) critical values		Narayan (2005) critical values	
		Low bound	Upper bound	Low bound	Upper bound
*F*_W_ = 11.12	1	4.99	5.85	6.43	7.51
	5	3.88	4.61	4.54	5.42
	10	3.38	4.02	3.77	4.54
Panel D: Long-run relation derived from *ARDL*(1,1,1)					
	*co2* = 62.61 −	15.25*gdp +*	0.93*gdp*^2^ −	0.04 *trend +*	*e*
		(4.80)	(0.07)	(0.02)	

${\chi}_{SC}^2$, ${\chi}_{ARCH}^2,\mathrm{and}\ {\chi}_{HETR}^2$denote Chi-squared statistics to test the null hypotheses of no serial correlation, no autoregressive conditioned heteroscedasticity, and no heteroscedasticity in the residuals; *JB*_*N*_and *F*_*FF*_ indicate Jarque–Bera and *F* statistics to test the null hypotheses of normal distribution and no functional misspecification respectively; *FW* is the *F*-value of testing the null hypothesis of ${\theta}^{\prime }=\sum \limits_{i=1}^1{\alpha}_i^{\prime }=\sum \limits_{i=0}^1{\beta}_i^{\prime }=\sum \limits_{i=0}^1{\gamma}_i^{\prime }={\delta}^{\prime }=0$ in the Wald test. Critical values are taken from the case of *unrestricted intercept and restricted trend*, 2 regressors and 30 observations (see Pesaran et al. 2001 and Narayan 2005). Probabilities are in brackets and standard errors are in parentheses. The pulse dummy variable of DP09Q1 is included in the estimation. Estimation period 1994–2013

We conduct the bound test of cointegration on *ARDL*(1,1,1). The sample value of *F*-statistic is greater than the upper bound critical values of Pesaran et al. (2001) at all three significance levels as tabulated in panel C. This indicates that the null hypothesis of no cointegration can be rejected meaning that there is a long-run relationship among the variables. As discussed in the econometric methods section, we also use the Narayan (2005) critical values for small sample bias correction. Evidently, the null hypotheses can be rejected even at the 1% significance level after small sample bias correction. Note that having cointegration from the ARDLBT supports the results of the Johansen method. Panel D reports the numerical values of the long-run relationship derived from *ARDL*(1,1,1).

Finally, we estimate numerical values of the long-run relationship between CO_2_ and income also using DOLS, FMOLS, and CCR as further robustness. Table 7 brings together the estimated long-run coefficients from all the five different methods.

**Table 7:** Long-run coefficients from the methods

Methods	*gdp*	*gdp*^*2*^	*Intercept*	*Trend*
	Coef. (std. er.)	Coef. (std. er.)	Coef. (std. er.)	Coef. (std. er.)
VEC	− 12.05** (4.13)	0.71** (0.24)	46.82** (17.66)	
ARDLBT	− 15.25*** (4.80)	0.93*** (0.29)	58.23** (27.24)	− 0.04* (0.02)
DOLS	− 15.96*** (4.95)	0.98*** (0.30)	60.40** (20.75)	− 0.05** (0.02)
CCR	− 19.21*** (3.68)	1.20*** (0.21)	72.39*** (15.83)	− 0.08*** (0.01)
FMOLS	− 19.40*** (3.33)	1.22*** (0.19)	73.15*** (14.31)	− 0.08*** (0.01)

The dependent variable is *co2.* The pulse dummy variable of DP2009 is significant in the FMOLS and CCR while insignificant in the DOLS as the first two have static structure and the last one has a dynamic structure. In DOLS, we set the maximum lag and lead being one, which is consistent with what we selected in the VAR/VEC and ARDL estimations and prefer Schwarz criterion to select an optimal ones. Estimation period covers 1994–2013

Coef., coefficient; Std. Er., standard error

*Indicates significance level at 10%

**Indicates significance level at 5%

***Indicates significance level at 1%

Clearly from Table 7, the coefficient of *gdp*^2^ is statistically significant across all these five methods. Meaning that we cannot reduce the quadratic functional form to the linear form. Additionally, *gdp*, as well as intercept and trend, are also statistically significant regardless of which methods’ results are considered.

Given that we have the small sample size, the estimated long-run coefficients from the different methods are quite similar to each other. For example, the coefficients on *gdp*^2^ are around unity. There is a consensus in the literature that ARDL estimations outperform all the rest alternative methods in the case of small samples (see Pesaran and Shin 1999; Pesaran et al. 2001 inter alia). Hence, we will use the ARDL estimation results in the next section.

## Discussion of the empirical results

This section discusses the results of the unit root and cointegration tests as well as the long-run estimations.

As can be seen in Fig. 1 and concluded from Table 3, CO_2_, income, and its powers all in per capita are non-stationary at their log levels while the growth rates of them are stationary. Interpretation of the non-stationarity is that mean, variance, and covariance of the levels of the variables change over time in the period selected. Moreover, any shock to them may have a permanent effect. Hence, one would have a difficulty in properly predicting future values of the CO_2_ and income using (log) levels of them. Oppositely, the stationary cases of the variables are mean reverting and, therefore, any shocks to them will have a temporary effect. Hence, the growth rates should be used in forecasting future values of the variables.

The Johansen test, and then the ARDLBT test as a robustness check, indicate that there is a cointegrated relationship between the emission and income. Being cointegrated implies that the variables share a common trend and move together in the long run. In other words, the emission and income variables are related to each other although they can deviate from this relationship in the short run. Main causes of these deviations are shocks that might be stemmed from policy interventions, fluctuations coming from the international and domestic markets, as well as changes in technological and institutional developments. Having cointegration between the emission and income also implies that the shocks to the relation that the variables establish in the long run are temporary and will be vanished out after some time.

As proposed by seminal studies, such as SB, we started with the cubic functional form to investigate the impact of income measures on CO_2_. This avoided any improper estimations and thus potential misleading in policy recommendation, which can be caused by misspecified functional form. The results from different cointegration methods showed that cubic term of the income was statistically insignificant in the estimations and when we excluded it the quadratic and linear terms of the income become more statistically significant. Therefore, we concluded that in Kazakhstan quadratic functional form represents the emission effects of income properly.

Table 7 presents that the coefficients of the quadratic term of the income are positive and statistically significant across all the five methods. This suggests “U”-shaped relationship between the CO_2_ and income in Kazakhstan. In order to make sure that this is the case in reality, a turning point of the income, in which the relationship turns from negative to positive, has to be calculated. We calculated the point using the ARDL estimation results.^11^ It was 8.22 for the period 1992–2013. This turning point value is lower than even the minimum value of the natural logarithm of income, which is 8.23 for the same period. In other words, the turning point is out of the sample values of the income.^12^ It has an important implication, which is that although we found “U”-shaped relationship between emission and income, in reality, the first half of the “U” can be ignored as the income value for the turning point is out of the sample period. Turns out that the relationship is monotonically increasing in reality, i.e., CO_2_ will increase as the income level will rise. We think that such kind of finding is relevant for Kazakhstan as the existing CO_2_ literature usually finds the EKS holds for the developed countries but usually not developing economies. In this regard, Kazakhstan is a developing/emerging economy that newly completed its transformation from the centrally planned economy to the market economy. Even a number of international institutions still consider Kazakhstan as a transition economy. Thus, this is a developing country and far away from being developed or advanced economy. Therefore, it has a long way to go in order to have such economic, institutional, and environmental development levels, in which income level can negatively associate with CO_2_. Besides, the monotonically increasing relationship between CO_2_ and income seems reasonable regarding the nature of the Kazakhstani economy: CO_2_ is highly associated with energy and the country is rich with energy resources and energy prices are heavily subsidized.

The estimated long-run coefficients of the linear and quadratic terms of income are reported in Table 7. Considering that some studies in the EKC literature interpret these coefficients as elasticities, and, thus, mislead readers, we would like to specifically highlight that the coefficients on linear or power term(s) of income are not elasticities. The elasticity has to be calculated as a partial derivative of the CO_2_ with respect to income. For elasticity formulas of different functional specifications, interested readers can refer to Gujarati and Porter (2009) and Hunt and Lynk (1993) among others.

Following the discussion in the previous texts, we calculated per capita GDP elasticity of per capita CO_2_ using ARDL estimation results. We calculated elasticities using minimum, mean, and maximum values of the per capita GDP over the period 1992–2013. The elasticities were 0.06, 0.93, and 1.94, respectively.^13^ Apparently, we found all the three elasticities to be positive. This finding is a numerical confirmation of our finding discussed previously postulating that the relationship between the emission and income is positive. If we consider mean elasticity, it shows that a 1% rise GDP causes 0.93% increase in CO_2_ both in per capita. It is noteworthy that such finding is consistent with the findings of earlier studies for Kazakhstan and similar countries. For example, Brizga (2013) found the elasticity being 0.86 for Kazakhstan using the Index decomposition analysis and OLS methods, which is different from our approach here. Additionally, Mikayilov et al. (2018) using cointegration method found it to be 0.70–0.82 for Azerbaijan, a country, which is very similar to Kazakhstan. This finding is also in line with the income elasticity of CO_2_ emissions found by Hasanov et al. (2018) for nine oil-exporting country cases, where similar economies of the region like Azerbaijan and Russia are included. Hasanov et al. (2018) found the elasticity to be 0.84 with consumption-based CO_2_ emissions and 0.54 with production-based CO_2_ emissions.

## Concluding remarks and policy recommendations

The current study was conducted considering the fact that there is no comprehensive time series analysis of the CO_2_ effects of income for Kazakhstan, an important country in Central Asia and CIS.

In order to avoid any improper estimations and thereby misleading policy recommendations, unlike many studies, we started our analysis with the cubic functional form as suggested by the seminal studies in the EKC literature. Also, to get more robust results, we used five different methods and addressed small sample size bias.

The empirical analysis showed that there is a long-run relationship between CO_2_ and income. Although “U”-shaped relationship appeared for the variables, the income value for the turning point of the relationship was outside of the sample period of 1992–2013. Hence, we concluded that the true impact of income on CO_2_ is monotonically increasing in Kazakhstan. In other words, EKC does not hold for Kazakhstan. We believe that such a conclusion is consistent with the socio-economic status of the country as it is a developing energy-rich economy. It will take a long time for Kazakhstan to have such a development level, in which income will decrease CO_2_. Numerically, we found that there is a one-to-one relationship between the variables in the long run.

We hope our research will be useful in making effective policy measures on CO_2_ in Kazakhstan. It concludes that the income has a positive impact on CO_2_. This finding suggests that environmentally friendly economic growth strategy should be taken into consideration in the future. Precisely speaking, if the strategy relies on heavy industry, such as oil, coal, and metal, then the country will get more pollution. In this regard, boosting economic growth in service and technology-related sectors would be preferable. Another policy measure to consider would be reducing the share of fossil fuels and achieve more share of renewables in energy generation. As a third measure, the Kazakhstani government can set up some regulations, such as high carbon tax, carbon capture, and emission trading schemes.

## Footnotes

1 The main weakness of the panel studies assuming homogeneity is that they constrained estimated coefficients to be the same across all members of the panel. Hence, they would not be able to capture the country-specific features of the relationship among the variables of interest

2 Brizga et al. (2013) employed the index decomposition analysis method for the individual countries in their panel. However, the output of the method is not conventional. For example, they do not report the long- or short-run equation as well as coefficients and elasticity for Kazakhstan.

3 For example, they do not report post-estimation test results and do not calculate the turning point of the income from the equation without energy consumption.

4 Although the authors mention in endnote 11 that they perform other cointegration methods, they do not report the estimation and testing results and do not compare them with those of ARDLBT.

5 Note that there are contrasting thoughts on using the power of a variable in unit root and cointegration tests. For example, Hong and Wagner (2008) mention that the square of per capita income does not have the usual linear unit root and cointegration distribution while many recent studies, however, consider powers as independent variables and use them in the unit root and cointegration testing.

6 If we go for the higher significance level, say 5 or 1%, then it will be concluded that the variables are trend-stationary, which is another form of non-stationarity, and, thus, a cointegration analysis can be still conducted.

7 We keep the deterministic trend in the VAR because excluding of it causes instability. The dummy variable takes unity in 2009 and zero otherwise, to capture the effect of the 2009 financial crisis. See Johansen (1995) and Juselius (2006) for the discussion of using deterministic variables including dummies in VAR and VEC modeling.

8 The cointegration test results in panel D do not change if we include the dummy variable in the test or exclude it.

9 Estimation results are not reported as they are not final results but they can be obtained from the authors upon request.

10 As we discussed in the “Theoretical framework and data” section, we include the deterministic trend in the estimation to see if it can exert any explanatory power.

11 Again, the reason why we preferred the ARDL is that it provides robust results in small samples compared to other alternative methods.

12 The minimum value of income is still 8.23 for the period 1994–2013 as well.

13 We also calculated the elasticities for the period 1994–2013. Only mean elasticity changed being 0.99 as the minimum and maximum values of the income are the same for 1992–2013 and 1994–2013.
